# Clinical Lecture on Tubal Abortion

**Published:** 1902-07-19

**Authors:** D. C. Rayner

**Affiliations:** Lecturer on Practical Midwifery in University College, Bristol. Assistant Physician Accoucheur, Bristol General Hospital.


### HOSPITAL CLINICS AND MEDICAL PROGRESS.

### CLINICAL LECTURE ON TUBAL ABORTION.

By D. C. RAYNER, F.R.C.S. (Eng.), Lecturer on Practical Midwifery in University College, Bristol. Assistant Physician Accoucheur, Bristol General Hospital.

ONE of the most interesting terminations of a tubal pregnancy is that in which the ovum, at an early period of its development, dies, and is extruded through the ostium abdominale of the tube into the peritoneal cavity, an event to which the term "tubal abortion" is applied. The presence of an impregnated ovum in the outer portion of the Fallopian tube usually leads to the occlusion of the abdominal ostium, and this is generally completed by the sixth or seventh week. So long as this orifice remains open, however, the ovum is in constant jeopardy of being expelled from the tube into the peritoneal cavity, and the nearer it is to the outer end of the tube the more likely is this to occur. When once the abdominal end of the tube has become closed the only way in which the ovum can escape is by rupture of the tube itself. We will consider briefly: First, the pathology of tubal abortion; secondly, the signs and symptoms; thirdly, the diagnosis, and lastly, the treatment. Tubal abortion is so often preceded by the formation of a "tubal mole" that it will be well for us to study the pathology of this interesting condition. An impregnated ovum, whether it develops in the tube or in the uterine cavity, is liable to a curious change, whereby it is converted into what is known as a mole. Moles arise only in the first few weeks following impregnation. If the ovum be examined during this time, its surface will be found covered with well-developed chorionic villi; the embryo will be enclosed in the amnion, while between the amnion and chorion a space exists which may be called the sub-chorionic chamber. As the embryo increases in size the amnion gradually encroaches on this space, and eventually obliterates it. Now a tubal mole is due to blood extravasated from the circulation of the embryo into this sub-chorionic chamber. Some observers do not agree with this view, but maintain that the mole is formed by the irruption of maternal blood into the embryonic membranes. That the former view, however, is probably the correct one is proved by an examination of the blood found in the subchorionic space. The blood of the embryo differs from that of the adult by the fact that the red corpuscles are nucleated, and microscopic examination of fresh tubal moles shows that the red corpuscles of the blood in the subchorionic chamber are nucleated. Of course in many cases tubal moles are found immersed in blood extravasated from the maternal vessels, but this does not alter the fact that the blood within the subchorionic chamber is derived from the fœtal vessels. The presence of this mole in the outer portion of the tube sets up contraction of its muscular coat, which results in its expulsion through the ostium abdominale into the peritoneal cavity, just as the presence of a mole in the uterine cavity excites contraction of the uterus which ends in its expulsion. In early uterine abortion the mole often fails to become completely detached from the uterine wall, and bleeding continues as long as it is retained. The same thing may happen in tubal pregnancy, and the mole so long as it is not ejected from the tube gives rise to recurrent hæmorrhage. This is the condition described as incomplete tubal abortion, and is perhaps more common than the complete form. During the early development of the ovum within the Fallopian tube it is important to remember that changes are taking place in the mucous membrane of the uterus, which result in the formation of a decidual membrane; and that with the expulsion of the ovum from the tube this decidual membrane is usually cast off from the uterus.

*Signs and Symptoms.*—The signs and symptoms in a typical case of tubal abortion are usually quite characteristic, and admit of little doubt as to what has occurred, while in other cases they are so indefinite as to render the diagnosis exceedingly difficult. In a typical case the patient, having gone a fortnight or three weeks over her period, is suddenly seized with severe pain in the lower part of the abdomen, accompanied with considerable collapse, and with signs of internal hæmorrhage, the symptoms are never so severe, however, as when the tube ruptures. With the onset of the attack there is usually a discharge of blood from the uterus, and in some cases a distinct decidual membrane is thrown off. On abdominal examination there is found to be considerable tenderness over the lower part of the abdomen, especially in one or other iliac region, and there may, if there has been a severe hæmorrhage, be signs of free fluid in the peritoneum. On vaginal examination the uterus will be found to be slightly enlarged, with perhaps some softening of the cervix. Within twenty-four or forty-eight hours after the onset of the attack it is often possible to detect a swelling behind and to one side of the uterus, this swelling being due to the coagulation of the blood that has escaped from the tube. If the case is one of "complete" tubal abortion the pain and other symptoms become less severe, and the swelling behind the uterus gradually disappears as the blood-clot becomes absorbed. If, however, the case is one of "incomplete" tubal abortion the symptoms tend to recur again and again until the ovum is extruded into the peritoneal cavity, and with each recurrence of symptoms the swelling behind the uterus will be found to have increased in size.

*Diagnosis.*—Although in a typical case of tubal abortion the diagnosis is easy, cases frequently occur which present considerable difficulty. Perhaps the condition that gives rise to symptoms most nearly resembling those of a tubal abortion is an early uterine abortion. In both cases the patient has usually missed a period, and is suddenly seized with abdominal pain, accompanied with uterine hæmorrhage. On careful examination, however, it is generally possible to distinguish between the two. In the first place, the pain in tubal abortion is usually more severe than in uterine abortion, and is situated on one side of the abdomen. The uterine hæmorrhage is rarely so profuse in tubal as in uterine abortion, and it is usually followed by the formation of a swelling behind the uterus. Then, again, if the uterine discharge be examined microscopically, the presence or absence of chorionic villi will be of great value in deciding whether the pregnancy is intra or extra-uterine.

*Treatment.*—The treatment of these cases depends to a great extent on whether the tubal abortion is "complete" or "incomplete," that is whether the ovum has been extruded into the peritoneal cavity, or whether it still remains partially attached to the tube. In the former case the hæmorrhage usually soon ceases; in the latter it continues as long as the ovum remains within the tube. As a general rule, seeing the imminent risk to the patient's life in all cases of extra-uterine pregnancy, it will be best to open the abdomen and remove the blood clot, and if the tube is much damaged remove it as well. Cases however in which only one hæmorrhage (and that not very severe) occurs, may be treated by palliative measures, viz. complete rest in bed, regulation of the bowels, diet, etc., in the hope that the blood may be absorbed and the parts recover.

